# Self-esteem depends on beliefs about the rate of change of social approval

**DOI:** 10.1038/s41598-022-10260-6

**Published:** 2022-04-22

**Authors:** Alexis An Yee Low, William John Telesfor Hopper, Ilinca Angelescu, Liam Mason, Geert-Jan Will, Michael Moutoussis

**Affiliations:** 1 Wellcome Centre for Human Neuroimaging, London, UK; 2 Paris Brain Institute, Paris, France; 3 Max Planck UCL Centre for Computational Psychiatry and Ageing Research, University College London, London, UK; 4 Research Department of Clinical, Educational and Health Psychology, University College London, London, UK; 5 Department of Clinical Psychology, Utrecht University, Utrecht, The Netherlands

**Keywords:** Human behaviour, Computational models

## Abstract

A major challenge in understanding the neurobiological basis of psychiatric disorders is rigorously quantifying subjective metrics that lie at the core of mental illness, such as low self-esteem. Self-esteem can be conceptualized as a ‘gauge of social approval’ that increases in response to approval and decreases in response to disapproval. Computational studies have shown that learning signals that represent the difference between received and expected social approval drive changes in self-esteem. However, it is unclear whether self-esteem based on social approval should be understood as a value updated through associative learning, or as a belief about approval, updated by new evidence depending on how strongly it is held. Our results show that belief-based models explain self-esteem dynamics in response to social evaluation better than associative learning models. Importantly, they suggest that in the short term, self-esteem signals the direction and rate of change of one’s beliefs about approval within a group, rather than one’s social position.

## Introduction

Computational modelling has made important contributions to the characterisation of self-esteem, the sense of one’s value or worth as an individual^1^. Low self-esteem predicts vulnerability to a range of psychiatric disorders, including mood^2,3^, anxiety^4^, and eating disorders^5^. Although very few studies have addressed the computational processes giving rise to self-esteem, important inroads have been made^6^. Computational modelling has refined the classic ‘sociometer’ theory of self-esteem^7^, confirming that self-esteem indeed varies with social approval, but it is surprises about approval, rather than its amount per se, which are most important. Yet current models of self-esteem contain key gaps. First, they are poorly connected to the phenomenology of beliefs about the self, and to clinical psychological theory of self-esteem, used in evidence-based therapies^8^. Second, they are silent about the involvement of rich belief-based, rather than associative, learning mechanisms^9,10^ (Table 1). Here we demonstrate cognitive mechanisms by which beliefs may inform self-esteem, elucidating how beliefs, formulated computationally, correspond to the abstract experience of feeling good, or bad, about oneself.

**Table 1:** Glossary of terms.

Term	Definition and Illustration
Belief (phenomenological)	The degree of subjectively experienced conviction in the truth of a statement. E.g. “I do not believe that ghosts exist”, “I believe that Mary has three children, but I’m not entirely sure”
Belief (mathematical)	Distribution of probability values about alternatives, e.g. p(Mary has less than 3 children) = 0.05, p(Mary has 3 children) = 0.8, p(Mary has > 3 children) = 0.15
Parameter	A quantity considered constant within a particular context or equation. E.g. how fast someone learns on average during an experimental session
Variable	A quantity that can vary in a particular context, e. g. our belief that Mary has 3 children may be updated by asking her
Learning	An updating of one’s model of the situation
Associative learning	Direct learning of the value of a state, e.g. of encountering a particular type of person, or an action, e.g. of making a choice
Inference	Updating beliefs within an existing model of a situation. E.g. ‘I inferred he must be English when I heard his accent’
Belief-based model	Model that uses observations to make inferences, rather than the goodness of outcomes to directly associate values

Clinically, beliefs are thought to have consequences. For example, negative beliefs about one's own worth may lead someone to withdraw from social interactions. These behaviours may then prevent patients from encountering disconfirmatory evidence (e.g. social approval), contributing to a vicious spiral^11,12^. A belief clinically associated with low self-esteem is one of global social disapproval (‘I will always be disliked when meeting new people’). Early developmental experiences, such as emotional maltreatment, can be thought of as ‘baking’ a set of assumptions about the self into the processing of beliefs and hence emotional processing^13^. This can have long-term maladaptive consequences e.g. believing that disapproval should be frequently expected. If these beliefs persist in new environments with contrasting evidence (i.e., when social support is present), mental health problems may ensue. Yet it is not at all obvious that beliefs are the best construct to understand the dynamics of self-esteem mechanistically. Brain algorithms corresponding to the experienced belief ‘if I speak to them, they’ll reject me’ may instead behave more like learning-theory associations^6^, or Bayesian inference^14^, or if–then, schematic thinking^15,16^. By modelling self-beliefs explicitly and comparing them with alternative algorithms, the processes which lead to low self-esteem and subsequent maladaptive behaviour may be more clearly understood. Understanding of belief dynamics may thus refine therapeutic treatments targeting maladaptive beliefs^17,18^.

**Figure 1:**
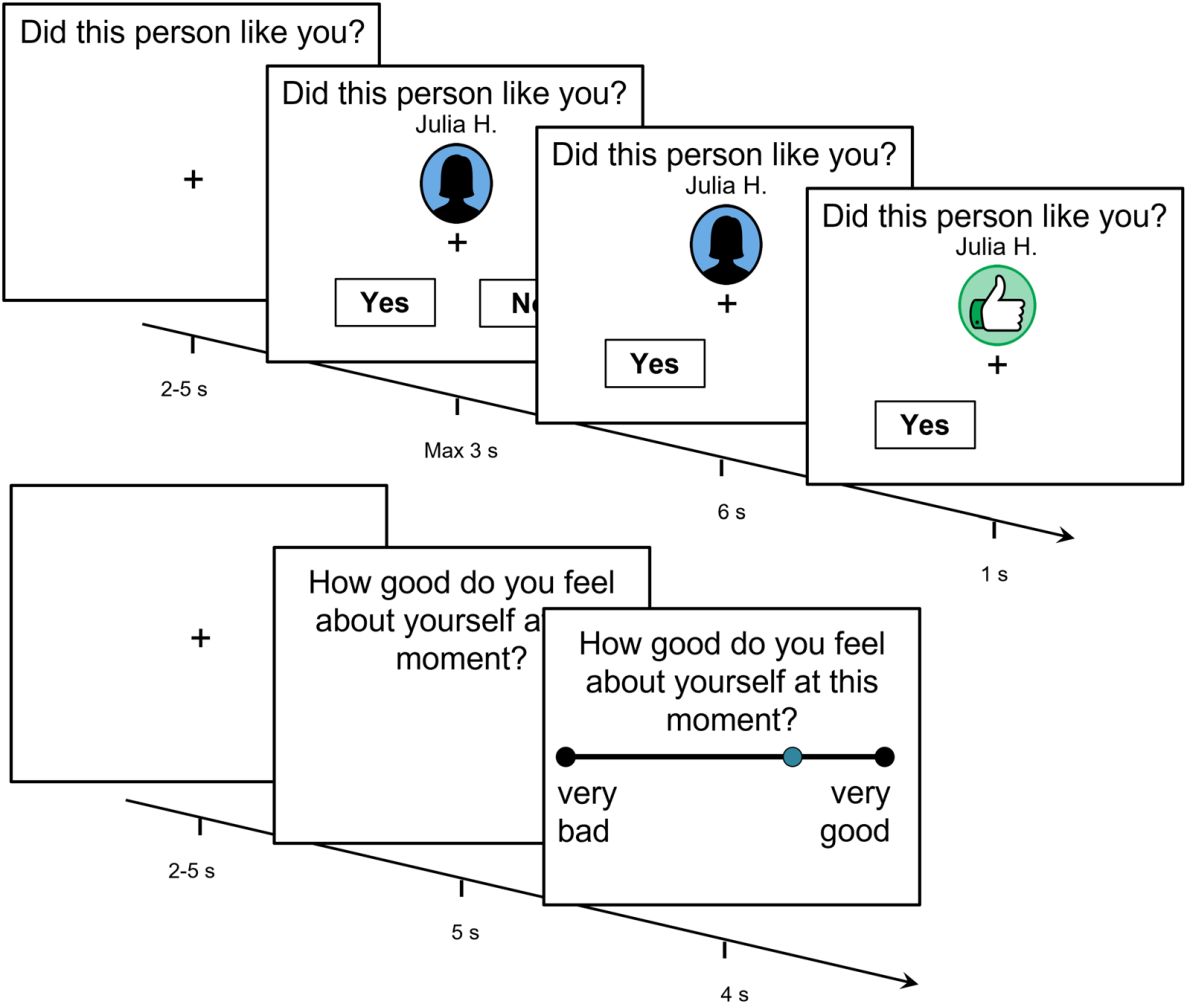
Trial structure of the task, reproduced with permission from Will et al.^6^. Participants were asked to predict whether another person liked them or not based on a cue indicating how approving a rater was in general toward other people. After the outcome become known, their self-esteem was probed by asking participants how good they felt about themselves. Please see “Methods” for detailed description.

In order to elucidate whether belief-based models offer the best account of momentary self-esteem, we first optimized our previous, associative models^6,19^ using data from an established task (Fig. 1). We compared these with belief-based models that drew on recent advances in the computational psychiatry of affect^20,21^. We first optimized associative models, to ensure that if belief models performed better, it was not simply because of features missing from the former. We investigated first, whether approval vs. disapproval have a different impact on learning^22^, and second, whether competence carries intrinsic reward (i.e., whether momentary self-esteem is shaped by both perceptions of social approval and competence in predicting if one will be liked (or not)^23^. Learning less from approval than disapproval may maintain negative beliefs about the self^17,21^. While it has been shown that differential learning rates occur in certain circumstances, it remains unknown whether they occur in situations of sequential evaluation, as might happen on social media or when one joins a new group. The mechanistic role of social competence (i.e., being able to predict others’ evaluations) is also uncertain, but may be important given that perception of non-social competence appears to carry intrinsic reward^23^. In addition, we explored a technical issue with current models, an inconsistency in mapping latent momentary self-esteem to behaviour leading these models to occasionally produce nonsensical momentary self-esteem ratings.

Importantly, we hypothesized that beliefs underpinning-esteem track the rate of change, of social approval. The rationale behind this rested on theoretical^18,20,24^ and empirical work^25,26^ implicating mood as signalling changes in the rate of reward. Knowing this rate of change, or ‘momentum’, allows past experience to better predict future outcomes by extrapolating across the time gap between the two. In the same way that mood-as-momentum is estimated through the history of prediction errors, momentary self-esteem may depend on recent unexpected social approvals or disapprovals (see Supplementary Material for more on the concept of momentum). The belief model used this concept in its core component which describes fluctuations in momentary self-esteem. Here, a Bayesian, affectively-laden belief which changed with social feedback mathematically represented the momentum of change in social approval i.e., how quickly things are improving or deteriorating socially. In real life, such beliefs would help guide what to do to improve social approval. In the context of a group such as a new social environment, where serial evaluations occur, we can thus refine the sociometer metaphor of self-esteem as a speedometer of beliefs about social approval—i.e. not just level of social approval but rate of change of social approval.

A belief-based model may explain momentary self-esteem better than an associative one for several further reasons. First, belief models capture uncertainty more sensitively than the associative models. Simply, uncertainty is inversely proportional to the amount of evidence accumulated. Uncertainty can then inform action variability. In contrast, sources of uncertainty in the associative models are fixed noise terms with no normative basis, empirically accounting for measurement error. Hence, belief models explain how new knowledge affects uncertainty, a key aspect of cognition, and how uncertainty may inform choice variability. Second, belief models naturally capture changing learning rates, as the more certain we are, the less our beliefs are shifted by a given outcome. Learning rates in associative models do not adapt like this.

To summarise, we developed a model of momentary self-esteem explicitly based on beliefs about approval within a group, and tested its superiority against optimised associative learning models^6,21^. We explored whether momentary self-esteem showed evidence for differential learning from approval versus disapproval, and whether perceived competence as well as approval were important factors influencing self-esteem. We then tested the winning belief model in a separate population, covering the entire range of trait self-esteem (i.e. including participants with clinically low levels of self-esteem). Our core hypothesis was that momentary self-esteem reflects beliefs in the rate of change of social approval. We found support for this hypothesis. Hence, momentary self-esteem may have a functional roles in predicting future social approval and guiding appropriate action.

Box 1: What is the difference between an associative and belief-based model of momentary self-esteem? The difference between the belief model and the associative model is that the former accumulates evidence about the self and the world into beliefs about what may or may not happen, whereas the latter directly accumulates an expected social value, i.e. how good or bad the situation is. To illustrate, the belief model can capture beliefs such as ‘I think 1 in 4 people in this group will like me’ by representing this via a so-called beta distribution. In this case, the beta distribution that captures this belief would have the parameters α = 1 and β = 3, which represent approval and disapproval counts respectively. On the other hand, the associative model would associate a single numerical value with each group, such as 0.75

## Results

Data was collected in an experiment involving serial evaluations from other people. Participants created an online profile by answering questions about their personality. They later performed a social evaluation task where on each trial, the participant had to predict if a different person appearing on the screen would approve or disapprove of them. Every second or third trial, they were asked ‘how good do you feel about yourself at this moment?’ via a visual analogue scale (see “Methods”). Three sets of participants were recruited in the context of a pilot study and two neuroimaging studies, on which we previously reported^6,19^. We compiled data collected in the pilot study and the first neuroimaging study into a ‘discovery’ dataset (n = 60) of healthy adults. The data from the second neuroimaging study served as a ‘confirmation’ dataset (n = 61), recruited from people who scored in the top and bottom 10% of trait self-esteem scores of the Rosenberg Self Esteem Scale of a community-representative sample^27^.

**Figure 2:**
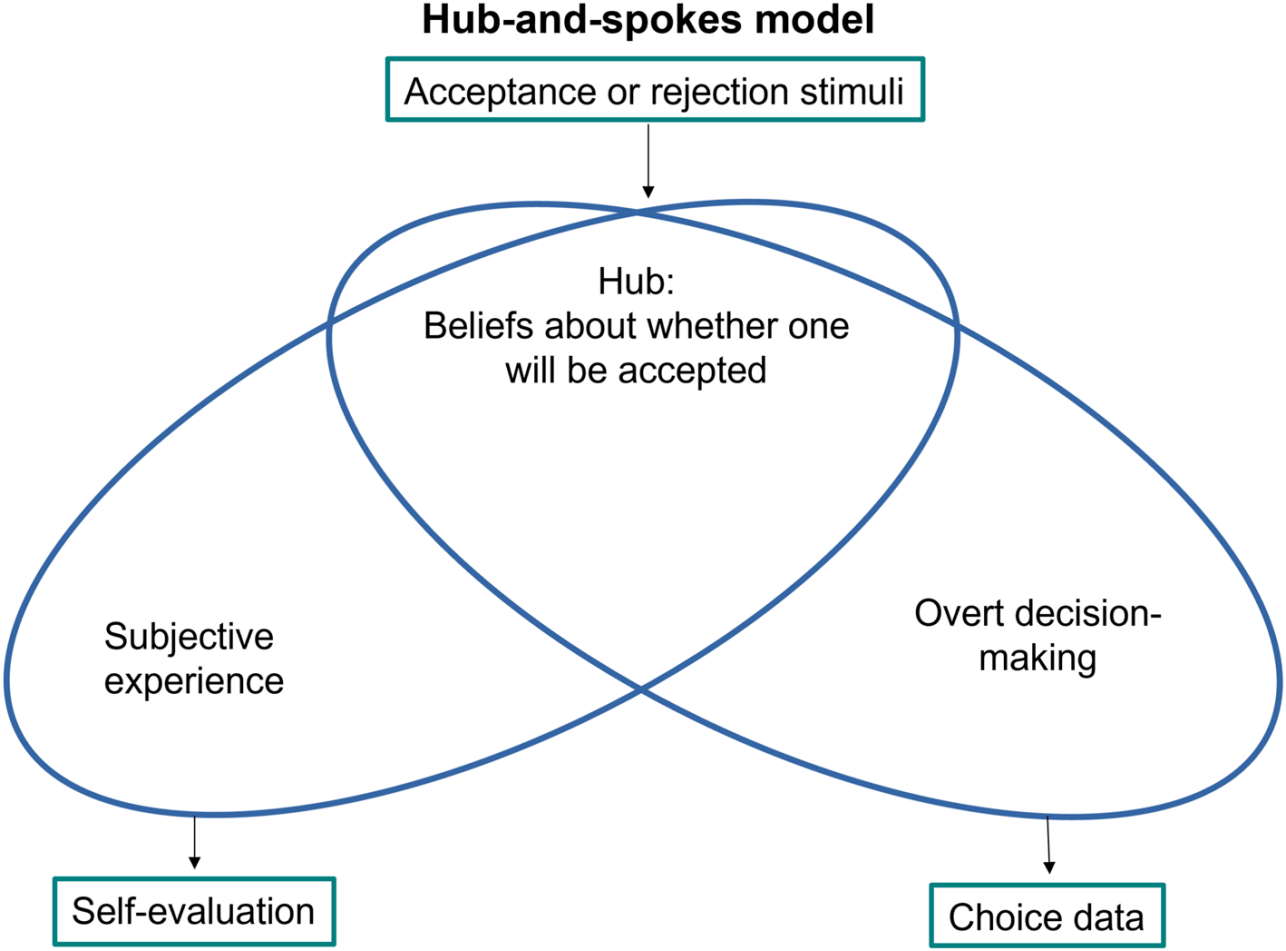
All models contained a ‘hub’ that learnt about approval by others, and so held expectations about whether one will be approved or not. The ‘hub’ contained probabilistic beliefs as illustrated or, in the case of associative models, approval values. Approval or disapproval stimuli then gave rise to (social) prediction errors (SPEs). Expectations and SPEs were passed on to ‘spoke’, or response processes: self-esteem (scale data) and approval predictions (choice data).

In the current study, we fitted a range of new computational models to the data to arbitrate between competing hypotheses about how momentary self-esteem is shaped by social feedback. The general form of these models is illustrated in Fig. 2. We first compared alternative forms of an established associative model to test if the “original” model was indeed the best model based on an associative process (see “Methods”, (1)–(6)). Here, people learned about the expected social value (ESV) of being accepted by different categories of others through associative learning. ESV then provided the basis for estimating Social Prediction Errors (SPEs), which determined momentary self-esteem. First, we compared models with differential learning from approval and disapproval with models with a single learning rate for both approval and disapproval. Second, we tested if including an elementary measure of social competence (i.e., `how well can I predict whether others like me?’) explained fluctuations in momentary self-esteem better than a model that solely includes a measure of social approval (i.e., `how much do others like me?’). Then, to test the key hypothesis of this study, we crafted a belief-based model. Here, beliefs about approval were explicitly formalised as beta distributions. We used the best associative model as a standard of comparison of how effectively the belief-based model explained the data.

### Investigating asymmetric learning rates

Across all participants, we found evidence against the hypothesis that participants learned differentially from approval vs. disapproval, in that models with such differential learning lost out in terms of parsimony, as assessed by the Bayesian Information Criterion^28^ (BIC) in both our datasets. We used as baseline our previous associative model^6^ wherein social-approval prediction errors (SPE) sum up to influence self-esteem (“Materials and Methods”, (1)–(6)). In all associative models, approval predictions were also subject to a ‘positivity bias’ parameter *B*, akin to optimism or perceived social desirability (5). We formulated differential learning from approval vs. disapproval by using different learning rates for these two outcomes (2LR models; (2)). As expected, models with 2 learning rates gave at least as good log-likelihoods as the baseline model, but lost out in terms of parsimony. Adding a term in (3) that depended on the expected value but not the prediction error (3b), or seeking greater parsimony by fixing the positivity bias *B* to be neutral for all participants, did not improve models. (Table 2, ‘separate term for expectations’ and ‘fixed positive bias’ respectively; see “Methods” for model details).

**Table 2:** Model comparison of baseline vs. two-learning-rate model for the discovery sample. The pattern of results was very similar for the test (subclinical) sample (see Supplement). The Bayesian Information Criterion (BIC) was used to compare the models, a lower BIC indicating a better fit^28^. Models were simultaneously fit to approval predictions and momentary self-esteem.

Model	Sum BIC	Mean BIC	Median BIC
Original	− **982.7**	− **16.1**	− **18.5**
2LR	− 926.3	− 15.2	− 17.4
2LR + separate term for expectations	− 479.4	− 7.9	− 16.0
2LR + fixed positivity bias	− 501.9	− 8.2	− 13.6
Competence-Acceptance (best, including separate expectation term)	− 374.8	− 6.1	− 8.8

BIC values of winning model are in bold.

### Investigating the role of perceived competence in momentary self-esteem in a social evaluation context

To investigate the possible role of momentary self-esteem boosting upon ‘getting predictions right’, we introduced terms similar to those of (3) and (3b) but with respect to success in prediction rather than approval (Box 3). We coded *Competence return* = 1 for correct predictions, *Competence retur*n = 0 for incorrect predictions, so that the ‘Competence Prediction Error’ (CPE) was the difference between the action-value for the chosen action and this return (8), analogous to SPEs. Thus, for mainly-disapproving groups competence PEs are opposite to social approval PEs, while for approving groups the opposite is true (of course only if people learn correct approval rates for the groups). We then added a CPE dependent term in the self-esteem equation, weighed by a free parameter *w*_3_ (10) (see “Methods” for more detail). In both the discovery and confirmation samples, the sum BIC deteriorated (Table 1). We note, however, that these competence analyses were exploratory, with the purpose of accounting for a potential contributor to self-esteem, as the task was not designed or powered to specifically investigate competence.

### The role of belief updating in momentary self-esteem

To test the hypothesis that momentary self-esteem is best understood as a belief based on integrating evidence from social feedback, we built belief-based models composed of three components. Here, a `hub’ updated beliefs about the probability of approval by each of the groups (Figs. 2, 5). The ‘prediction’ or choice spoke was similar to the associative models, except instead of expected value, choice was based on expected probability of approval. The self-esteem ‘spoke’ accumulated social approval prediction errors, trial by trial (see “Methods” and Box 4).

In the belief-based formulation we replaced the key sum-of-PEs term of (3) with an estimate of the belief about how fast the probability of approval was changing at any particular trial. Then, rather than the baseline Self-Esteem (*w*_0_ in (3)) and Gaussian noise (4) of the established model, we passed the ‘speed of approval change’ belief distribution through a sigmoid response function. This offered a more self-consistent approach than the Gaussian noise model, whose outputs are not confined to the bounded response interval participants used. Sampling from this sigmoid-transformed distribution naturally provided variability to the self-evaluation choices, so that additional noise terms were not needed, in accordance with sampling-based decision-making approaches^29,30^.

We first carried out model comparison of variants of the belief model in the ‘discovery’ sample to compare it to the established associative model and an associative_sigmoid_ model. In the latter, we equipped the associative model with a sigmoid response function to make it more comparable to the belief-based model. We found that the belief model outperformed the other 2 models (Table 3). This led us to hypothesize that it would also be the most successful out-of-sample, in the confirmation dataset. This was indeed the case (Table 4), providing evidence that self-evaluation is best thought of as depending on beliefs about the rate of change of approval, rather than an average error of associatively-learnt value.

**Table 3:** Comparisons between the belief model, the associative model, and the associative model with sigmoid response function (assoc_sigmoid_) in the discovery dataset. The belief model won the comparison. In this sample, participants were recruited from university databases.

Model	BIC		Approval predictions sum log likelihoods		Self-esteem rating sum log likelihood density		BIC_belief_ -BIC	
*Statistic*	Median	Mean	Median	Mean	Median	Mean	Median	Mean
Belief	− 24.2	− 121	− 54.1	− 59.9	96.6	146	0	0
Associative	− 19.3	− 19.0	− 53.3	− 59.0	92.3	93.6	− 4.9	− 102
Assoc_sigmoid_	9.4	− 4.2	− 53.4	− 59.8	81.8	87.0	− 33.6	− 117

**Table 4:** Out-of-sample replication of model-comparison (‘confirmation’ sample).

Model	BIC		Approval predictions sum log likelihoods		Self-esteem rating sum log likelihood density		BIC_belief_ -BIC	
*Statistic*	Median	Mean	Median	Mean	Median	Mean	Median	Mean
Belief	− 53.7	− 292	− 56.5	− 59.4	104	230	0	0
Associative	− 39.4	− 26.1	− 52.9	− 57.5	89.1	95.4	− 14.3	− 266
Assoc_sigmoid_	8.6	11.1	− 55.6	− 58.9	70.1	78.2	− 61.7	− 303

By conventional BIC criteria, there is very strong evidence for the belief model outperforming other models for both datasets. We provide median BIC values, which lead to similar conclusions, as they are less sensitive to outliers. The associative_sigmoid_ model performed worse than the original associative model itself, showing that the belief model’s better performance was not driven by the response function alone—hence, the belief dynamics appear crucial to the winning models.

**Figure 3:**
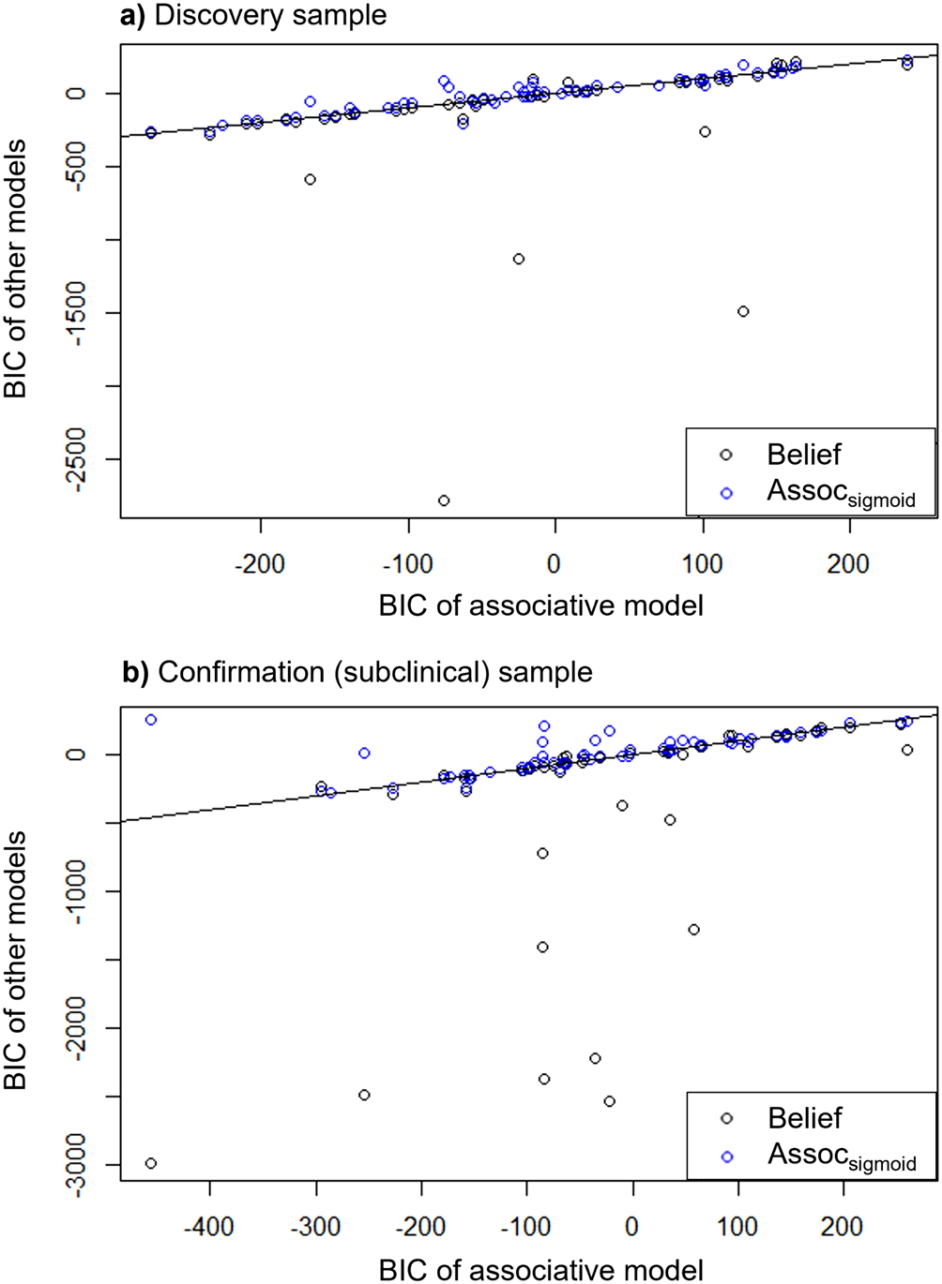
Participant-by-participant model comparison, each dot representing one participant (Discovery n = 60, Confirmation n = 61). The x-axis is the BIC score when fitted to the associative model, and the y-axis is the BIC value when fitted either the belief or associative with sigmoid response function models. The straight line represents equal BIC when fitted with the associative model.

**Figure 4:**
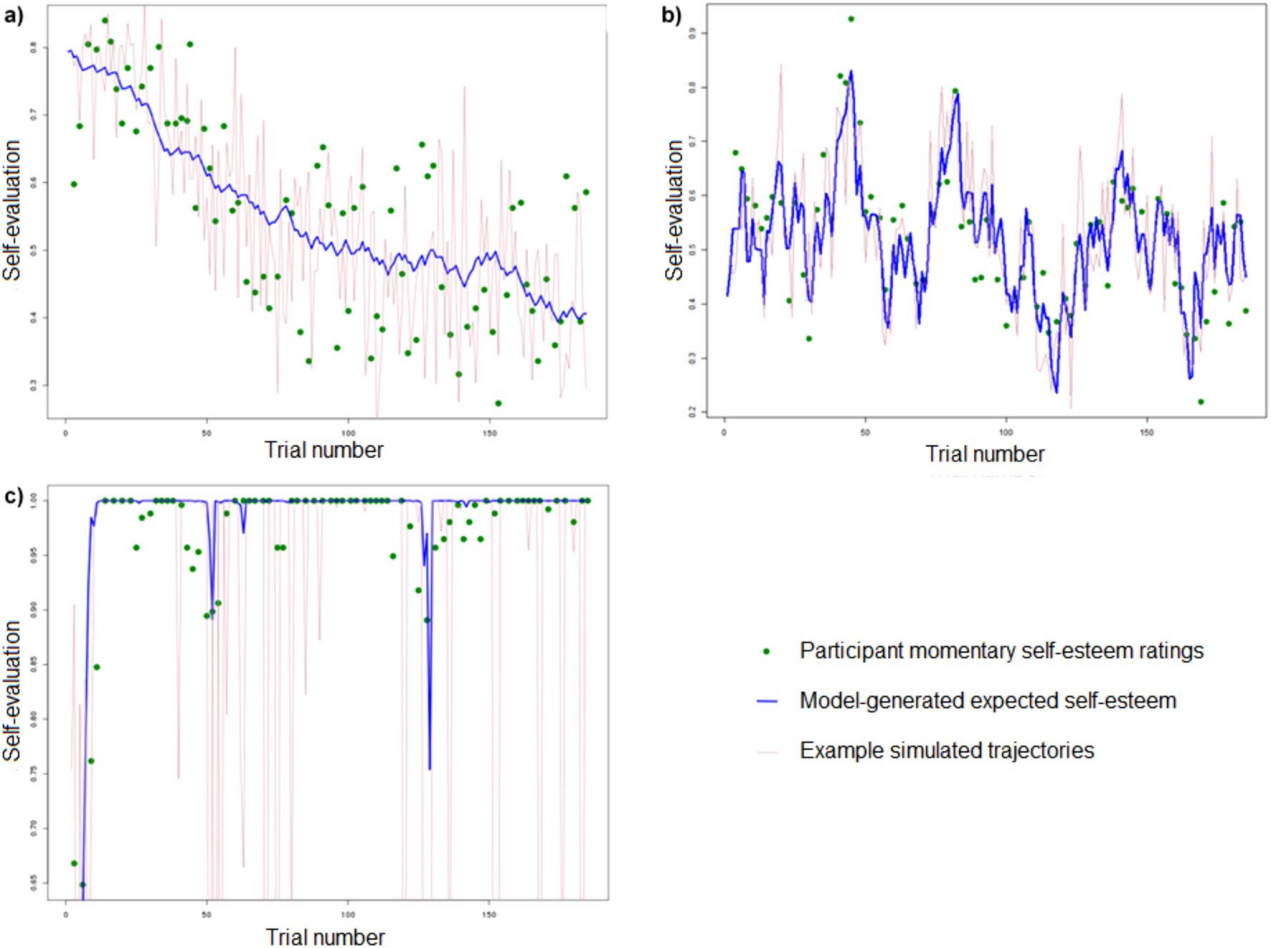
Self-esteem plots for three participants. Correlations between model-produced expected self-esteem values and actual self-esteem ratings were high for all 3, at 0.744, 0.858 and 0.818 respectively. (**a**) Example of gradual change in self-evaluation, in the context of noisy responding (**b**) SE following rather precisely the changes in recent approval and disapproval (**c**) ‘Brittle’ high SE. These patterns are seen in participants across both datasets.

To further understand the results, individual participants’ BIC scores were plotted for each model Fig. 3). This showed that the belief model performed at least as well or slightly better for most participants, but greatly outperformed the others for about 10% of the entire sample. To illustrate why the belief model performed well, we show examples of fits in Fig. 4. The model was able to capture a striking diversity of self-esteem patterns with different underlying narratives. While the model fits the participant in Fig. 4A by capturing gradual change over many trials, as well as giving a good account of choice variability (red line), in Fig. 4B it captures social prediction-error driven trial-by-trial fluctuations, while in Fig. 4C it shows ceiling behaviour characterized by brittleness, that is, sudden drops in response to selected social disapproval feedback stimuli. This indicates that the model’s key hypotheses are valid across different behaviours and participants.

## Discussion

We aimed to deepen understanding of how social feedback shapes momentary self-esteem in the context of multiple brief encounters, as might happen when one enters a new social milieu. We found that feedback from others is incorporated into beliefs, not only about current levels of approval, but the rate of change of approval by members of a group. The underlying beliefs can be seen as assumptions about the self, and how likely the world is to accept us (self-schema^31,32^). Dynamic belief updates then drive changes in self-esteem. By formalising beliefs as beta distributions, we characterized how changeable beliefs were– the narrower the beta distribution, the more precise or fixed the belief, and the more difficult it is for evidence to shift it. Finally, in a number of additional analyses, we found that being able to accurately predict social approval or disapproval was generally not prioritised in this context. Neither was differential learning from approval and disapproval a major factor, unlike in other qualitatively comparable situations, which use repeated feedback from few raters^17,21^.

Explicitly modelling belief representation is important, as it allows self-esteem to be linked to cognitive models of affective disorders, which emphasise the role of negative beliefs in the generation and maintenance of emotional disorders^33,34^. Belief-based models finesse the associative learning framework, helping quantify phenomenological beliefs about self-esteem within existing cognitive and associative accounts of behaviour. Such models help delineate how far phenomenological beliefs correspond to Bayesian beliefs—a relation which is substantial, but certainly not perfect^29^.

What features drove the improved performance of belief models? They differed from associative models in two key ways: one, they naturally used belief uncertainty to weigh the magnitude of belief updates, and also to drive response variability, as approval or disapproval has a greater effect if beliefs are more uncertain; and two, they used a sigmoid response function. To test which one of these most improved performance, we built a version of the associative model which also had a sigmoid response function. This failed to provide improved fits. However, the theoretical consistency and the success of a sigmoid response function mapping underlying beliefs onto self-reports may be of broad usefulness in computational psychiatry, as many experiments rely on continuous, bounded scales like the one in this task^35,36^.

Our finding that momentary self-esteem tracked the rate of change of approval is consistent with recent computational models of mood as momentum^20,37^. In such models, mood signals not how rewarding the current environment is, but how much the environment is improving or worsening. This may be beneficial in enabling to quickly adapt behaviour to changes in the environment, though over-reliance on such signals may give rise to mood instability^26^. Tracking the rate of change in approval may be similarly adaptive for social functioning and safety when encountering new groups. We predict that, as in the mood-as-momentum model, excessive sensitivity to changes in social approval may be maladaptive and give rise to mood symptoms via inaccurate beliefs about the self, for example in emotionally unstable personality conditions.

The success of belief models further finesses the Sociometer Hypothesis^7^. It is concordant with our previous interpretation of momentary self-esteem being akin to a read-out of recent changes in one’s social standing^6^, but now the useful quantity that the sociometer may measure has become clearer. The accumulation of social prediction errors into self-evaluation may construct beliefs about the temporal evolution of approval, more like a speedometer which captures how fast we move forward. In real life, momentary self-esteem could serve as an indicator of whether one’s recent, socially relevant behaviour is successful. Recent work suggests that mood-as-momentum may explain further psychological features of mood, such as its dependence on counter-factual information, and may feed into decision-making by estimating the added value of recent vs. average behaviour, known in reinforcement learning as ‘advantage’^37^. This naturally suggests how our account of self-esteem may be further tested and applied to interpersonal decision-making.

Model comparison suggested that people did not learn differentially from approval and disapproval, against our hypothesis^17,22^. The simplest explanation is that here positive and negative prediction errors were just as salient, unlike in non-social settings using electric shocks^38^, or in social settings where the immediate environment is less safe than the laboratory, or in situations where no information is provided about raters and so learning is even more prominent. Approval and disapproval experienced in front of a group of people may be especially important for those high in social anxiety^21^. The present work focuses on mechanisms of momentary self-esteem beliefs in general, but future studies should examine whether differential updating depends on self-schemas associated with lower self-esteem^17^, or differs depending on how threatening an environment is. Next, we did not find that predicting approval or disapproval (social competence) contributed to momentary self-esteem. On the one hand it is reassuring that we specifically assessed the influence of approval on self-esteem, but we must guard against naively concluding that competence is of no importance in social settings, as it appears to be intrinsically rewarding in non-social settings^23^. It would be interesting for future studies to clarify the contributions of competence and approval to the computation of self-esteem across individuals and states of clinical importance, as both competence and approval are likely to have adaptive roles^39^. Indeed, self-esteem may depend idiographically on different competencies in different individuals.

A strength of this study is that the winning model was optimized in one dataset but replicated in another, which included a different, wide range of trait self-esteem. This allowed out-of-sample verification of the model’s robustness, but also reinforced its relevance. First, it is generalizable to the full range of trait self-esteem found in the community. Second, it adds confidence to its use in clinical research, as the bottom decile of trait self-esteem included in our replication sample have a higher risk of psychiatric disorders^2,4,5^. In terms of limitations, both samples were restricted to young adults (mean age = 20.7, SD = 2.7), while self-esteem is important throughout the lifespan and cannot be assumed to follow the same dynamics. However, important aspects of self-esteem such as its dependence on one's peers do remain stable throughout one’s lifetime^40^, lending some confidence to the generalizability of our findings here.

In terms of clinical implications, the dynamics of self-esteem described by the belief model may have potential as transdiagnostic targets for therapeutic interventions, given the high co-morbidity and putative mechanistic similarity between disorders involving self-esteem^41,42^. As the model enables inferences about beliefs, it can inform cognitive behavioural therapy, where beliefs are explicitly inferred and analysed, for example by specifically targeting belief-updating^33,34,43^. Model parameters can guide personalised therapies as they are individual-specific. On exploration of appropriate thresholds, the model may also identify vulnerabilities or act as a diagnostic tool. Group-level inferences can also support interventions such as psychoeducation^44^.

In conclusion, we demonstrate that self-esteem depends on beliefs about how fast one’s social approval is changing. This enables key connections with the rich literature on beliefs, clinical psychological theory of affect, and decision-making studies, shedding light on the fundamental processes on which our view of ourselves is built on—potentially paving the way towards novel therapies which lessen the self-destructive consequences of low self-esteem.

## Materials and methods

### Participants

We first used a `discovery sample’ consisting of 60 participants (mean age = 20.8, SD = 2.14, 34 female), recruited from University College London volunteer pools. These included 39 participants (26 female) who gave valid data in a neuroimaging study^6^ plus 21 participants who performed the task in the context of a behavioural study. The University College London Research Ethics Committee approved the study (ID Number: 3450/002). A subsequent ‘test sample’ consisted of 61 participants (mean age = 20.6, SD = 3.2, 32 female), who were selected from a larger database of the ‘Neuroscience in Psychiatry Network Project’ so as to have Rosenberg Self-Esteem Scores either in the top (31 participants) or the bottom (30 participants) decile of an epidemiologically representative population^19,45^. All participants gave informed consent. This study was approved by the London-Westminster NHS Research Ethics Committee (number 15/LO/1361). All methods were performed in accordance with the relevant guidelines and regulations. The reader is referred to the published protocol^45^ and related studies^6,19^ for further details. Exclusion criteria for all datasets included lack of fluency in English, colour blindness and current psychiatric disorder. Scanned samples also had as exclusion criteria neurological disorder, brain injury, and left-handedness.

### Experimental task

Seven days before the task, the participants were told to submit a profile of themselves which would be uploaded to an online database. This consisted of answers to personal questions, and they were told that people would decide based on it whether they would like to be friends.

During the task, participants received social approval or disapproval feedback from 192 strangers in the “discovery sample” (half of them of female-typical outline) and 184 in the “test sample”. The participants were told that raters were divided into four groups based on overall likelihood of approving of others. On each trial (Fig. 1), the participant is shown which group the rater is from. Then, the participant is asked to predict the feedback. 6 s later, actual feedback was revealed via a thumbs-up or thumbs-down icon. 24 trials with no feedback were included in the samples that underwent scanning. Every 2–3 trials, participants are asked ‘how good do you feel about yourself at the moment?’ on a visual analogue scale anchored at ‘very bad’ and ‘very good’.

Unknown to participants, ratings were in fact computer-generated, such that they received 50% approvals and 50% disapprovals each, and the approval probability across four groups was approximately 15%, 30%, 70% and 85%, mirroring feedback received. The number of trials differed slightly between data-sets, as did the exact proportions of approval or disapproval per rater group, but these aspects were irrelevant to the present modelling study.

### Associative (Rescorla–Wagner based) reinforcement learning models

For all models in this paper, i.e. the associative model, the competence-acceptance model, the two-learning rate model, and the belief model, Supplementary Table S4 provides the symbol, range and definition of every parameter. It is recommended that this section is read alongside Table S4. The original model was described in Will et al. 2017, and we summarise it here, with the relevant equations in Box 2 below.

According to the Rescorla–Wagner (RW) rule, values are updated by ‘surprisingness’ coded in the form of prediction error^46^. This inspired the original model, where the momentary self-esteem in a given trial was calculated by taking the previous trial’s self-esteem and adding a term proportional to prediction error i.e. the difference between outcome and expectations about social approval or disapproval.

The use of this rule was then formalised in two ways. First, as different people incorporate prediction errors into their beliefs at different rates, the social prediction error (SPE) must be differently weighted for each participant, as indicated by w_1_ in (3), which has a different value for each participant. Second, because memory decays over time and older observations become replaced by newer ones, prediction errors from previous trials decayed on every trial; prediction errors were multiplied by a decay parameter *g* between 0 and 1 (3).

The trial-by-trial self-esteem value above represents state-like self-esteem, a component which reflects momentary changes in the psychological *state* in response to recent social approval or disapproval^47^. However, self-esteem has another component: *trait* self-esteem, which is relatively stable across time and situations^40^. To account for this, a baseline self-esteem term was linearly added. It represented self-esteem which remained stable in the experiment. In this way, the model captured both relatively stable and changeable self-esteem.

Then, to allow the model to generate noisy real-life data, a Gaussian noise term was added, as per (4).

Box 2. Equations of associative models. For all equations in this paper, see Supplementary Table S4 for more detailed definitions of free parameters as well as their numerical ranges. **‘Hub’ **equations: Rescorla–Wagner learning of Expected Social Value (ESV), which is then used to calculate Social Prediction Error (SPE).

$${\text{S}}PE^{\left( t \right)} = R_{k}^{\left( t \right)} - ESV_{k}^{\left( t \right)} \quad \quad \quad {\text{Equation}}\;\left( 1 \right)\;{\text{Social}}\;{\text{Prediction}}\;{\text{Error}}$$

where SPE is the Social Prediction Error upon receiving a return of R = 0 if ‘disapprove’ or R = 1 if ‘approve’ from rater group k, and t is trial number

$$ESV_{k}^{{\left( {t + 1} \right)}} = ESV_{k}^{\left( t \right)} + \eta_{c} SPE^{\left( t \right)} \quad \quad \quad {\text{Equation}}\;\left( 2 \right)\;{\text{RW}}\;{\text{update}}$$

Terms are as above. Note that the learning rate η is subscripted to denote that in some models, it might be different for c = approval vs. disapproval

Self-esteem ‘spoke’:

$$SE^{\left( t \right)} = w_{0} + w_{1} \mathop \sum \limits_{j = 0}^{t} \gamma^{t - j} SPE^{\left( j \right)} + \epsilon \;{\text{Equation}}\;\left( 3 \right)\;{\text{momentary}}\;{\text{self-esteem}}$$

where SE is momentary self-esteem, $w_{0}$ is baseline self-esteem, $w_{1}$ is the weighing factor for the prediction error term, γ is the forgetting factor which controls the decay of effect of previous feedback on self-esteem, and $\epsilon$ is a Gaussian noise term.

$$\Delta SE_{EV} = w_{EV} \mathop \sum \limits_{j = 0}^{t} \gamma^{t - j} ESV^{\left( j \right)} \quad \quad {\text{Equation}}\;\left( {3{\text{b}}} \right)\;{\text{Separate}}\;{\text{expectation}}\;{\text{term}}\;{\text{for}}\;{\text{SE}}$$

where w_EV_ is a weighting factor for a separate expectations term

$$\epsilon \sim N\left( {0,\sigma } \right)\quad \quad \quad \quad \quad {\text{Equation}}\;\left( 4 \right)\;{\text{SE}}\;{\text{Noise}}$$

Prediction of approval policy, or **choice ‘spoke’**:

$$Q_{k} = ESV_{K} + B\quad \quad \quad \quad {\text{Equation}}\;\left( 5 \right)\;{\text{Action}}\;{\text{value}}\;{\text{for}}\;{\text{predicting}}\;{\text{`}}\;{\text{accept}}\;{\text{'}}$$

where B is a bias term

$$\pi_{k } \propto exp\left( {Q_{k} /\tau } \right) \quad \quad \quad \quad {\text{Equation}}\left( 6 \right)\;{\text{Probability}}\;{\text{of}}\;{\text{predicting}}\;{\text{`}}\;{\text{accept}}\;{\text{'}}$$

where $\tau$ is decision temperature

### Modified associative models

#### Separate term for expectations

Here, we expanded the description given in Box 2. Will and colleagues^6^ tested whether the expected social value (ESV) of approval had an additional effect on changes in momentary self-esteem above and beyond their effect captured by the SPE term by modifying (1) to include a separate additive expectation term (3b). Versions of both the 2LR and CA models including this term were compared (See Table 1).

#### Fixed positivity bias

The original associative model also included a positivity bias which represents an individual’s willingness to predict being liked even when evidence suggests otherwise. Individuals with a larger bias, and thus who are more “socially optimistic”, would continue to predict approval from groups for whom they had a negative ESV. To justify its inclusion in both the 2LR and CA models, we compared them with versions where the bias was fixed to the same, neutral value for all participants.

#### Valenced learning rates

We tested a version of the original model that included two learning rates, h_c_ in Box 2. This was implemented as follows:

7 $$\begin{aligned} ESV_{k}^{t + 1} & = ESV_{k}^{t} + \eta_{pos} SPE^{t} for\,\, SPE > 0 \\ ESV_{k}^{t + 1} & = ESV_{k}^{t} + \eta_{neg} SPE^{t} for\,\, SPE < 0 \\ \end{aligned}$$

This allows for the possibility that individuals update their expectations about approval differentially depending on the valence of the SPE.

#### Competence-acceptance model

This model depended on Competence Prediction Errors, based on Competence action-values. The latter assessed how competent participants regarded their own ability to correctly predict ‘approval’ or ‘disapproval’ (which could be inferred through checking whether their prediction matched actual feedback). The competence equations are shown in Box 3 below. Because in the experimental task participants were asked to predict whether they would be approved or disapproved of before receiving the actual feedback, competence is defined here as whether the participant correctly predicted whether they would be approved or disapproved of (9).

Competence-related self-esteem then depended on (unexpected) prediction success. Whereas SPEs capture the difference between how much a participant expected a rater to like them and how much that rater actually liked them, Competence PEs capture the difference between how much a participant expected to correctly predict feedback and whether they actually correctly predicted feedback. Competence was incorporated in SE updates as per (10) (Box 3). Here, the weighting factor, *w*_*3*_, dictates to what extent momentary self-esteem is dependent on the competence of the participant versus their approval by others, with a value of 1 meaning solely dependent on approval and a value of 0 meaning solely dependent on competence.

Box 3. Competence equations $Q_{a} \left( t \right) = ESV_{k} ^{{\left( t \right)}} \;for\;a = accept$

$$Q_{a} \left( t \right) = 1 - ESV_{k} ^{{\left( t \right)}} \;for\;a = reject\quad \quad {\text{Equation}}\;{\text{(8)}}\;{\text{Competence}}\;{\text{action - values}}$$

$$CPE^{{\left( t \right)}} = Competence^{{\left( t \right)}} - Q_{a}^{{\left( t \right)}} \;{\text{Equation}}\;\left( 9 \right)\;{\text{Competence}}\;{\text{Prediction}}\;{\text{Error}}$$

where Competence = 1 if participants were correct and Competence = 0 if participants were incorrect in their predictions about social approval

$$SE\left( t \right) = w_{0} + w_{1} \left( {w_{3} \mathop \sum \limits_{j = 1}^{t} \gamma^{t - j} SPE_{j} + \left( {1 - w_{3} } \right)\mathop \sum \limits_{j = 1}^{t} \gamma^{t - j} CPE_{j} } \right) + \epsilon \quad \quad {\text{Equation}}\;\left( {10} \right)\;{\text{Self-Esteem}}\;{\text{based}}\;{\text{on}}\;{\text{competence}}\;{\text{and}}\;{\text{approval}}\;{\text{PEs}}$$

#### Sigmoid response function

The associative_sigmoid_ model was a modified version of the original associative model where the baseline self-esteem term and weight on social prediction errors (SPE) term were replaced by a sigmoid response function, where SE_state_ is represented by SPEs summed over preceding trials, as in the associative model. As with the belief model, *m* and *B* represent the sensitivity of self-esteem changes and the participant’s shift or bias respectively. This was to allow for better comparison between the two models, as the belief model below also uses a sigmoid response function:

11 $$SE = \frac{1}{{1 + e^{{ - \frac{{SE_{state } + B}}{1/m}}} }}$$

where m represents sensitivity, B bias, and SE_state_ = $\sum\nolimits_{j = 0}^{t} {\gamma^{t - j} } SPE^{\left( j \right)}$ term from (3).

### Belief models

The model represents social approval beliefs in the form of a beta-distribution—in other words, a ‘beta-belief’. This distribution was used due to several reasons. First, it allows for the representation of prior beliefs, as participants are likely to have beliefs about their social approval before the experiment. Second, certainty of belief can be coded, such that stronger beliefs built on more evidence are more precise—and thus harder to shift. Third, such a model is simply updated as new information accumulates and beliefs are updated. How this evidence accumulation operates is explained in Box 4 below.

**Figure 5:**
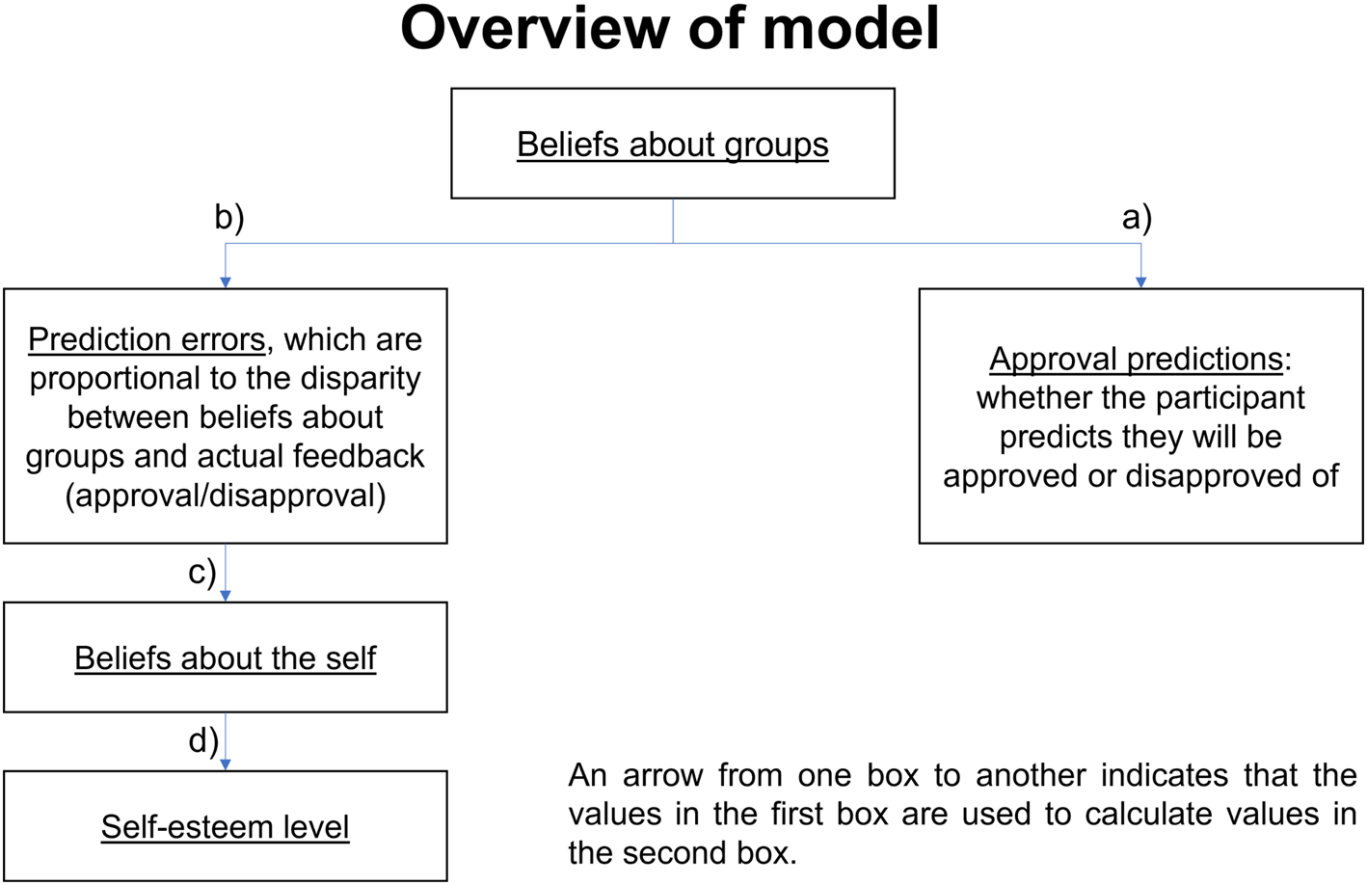
Overview of the belief-based hub-and-spokes model (see also Fig. 2).

The model is illustrated in terms of the ‘hub and spokes’ scheme in Fig. 5. As the central belief distribution about approval gets updated, prediction errors are created. These prediction errors are summed over the trials to form beliefs about one’s overall approval. A second belief distribution is formed by accumulating evidence of these PEs. Again, this is subject to ‘leaky’ accumulation of evidence, as described in Box 4. Finally, a psychometric sigmoid response function is applied to the distribution, calculating predicted self-esteem. Two types of beliefs are hence mathematically represented: beliefs about specific groups, and beliefs about the self.

Box 4. Accumulating evidence towards forming beliefs about the self in belief-based models Imagine that we accumulate evidence about a particular quantity *X* by remembering only a fraction g < 1 of it, and adding an update d. Consider three update steps:

$$\begin{aligned} X_{t } & = X_{t - 1} \gamma + \delta_{t} = \left( {X_{t - 2} \gamma + \delta_{t - 1} } \right)\gamma + \delta_{t} \\ & = \left( {\left( {X_{t - 3} \gamma + \delta_{t - 2} } \right)\gamma + \delta_{t - 1} } \right)\gamma + \delta_{t} \\ & = X_{t - 3} \gamma^{3} + \left( { \delta_{t - 2} \gamma^{2} + \delta_{t - 1} \gamma + \delta_{t} } \right) \\ \end{aligned}$$

We see that this quantity takes a very similar form as the key sum in (3), a sum of prediction errors weighted by increasing powers of g. Using a learning rule of the original form at each step makes it explicit that we are dealing with learning or inference, and obviates the need to encode the exponential kernel of the established, associative model ‘by hand’. To move from a value-setting to a belief setting, we accumulate separately the ‘wins’ (positive PEs) and ‘losses’ (negative PEs), and use them to parametrize a beta-belief distribution *Beta*(*a*_*t*_^(S)^*, b*_*t*_^(S)^). We will use a slightly more complex update rule, to make sure that α and β cannot drop below 1 and to allow calibration of the impact of the PEs through a parameter w. Furthermore, this formulation invites the interpretation that what we are dealing with is beliefs about a quantity proportional to the average difference between expectation and return, in essence a measure of the gradient or momentum of how fast the quantity to which d pertains, i.e. social approval, is changing:

12 $$\begin{array}{*{20}l}{\alpha _{t}^{{(S)}} = \left( {\alpha _{{t - 1}}^{{(S)}} - 1} \right)m + 1 + w\;\max {\mkern 1mu} (\delta ,\;0)}\, \hfill \\ {\beta _{t}^{{(S)}} = \left( {\beta _{{t - 1}}^{{(S)}} - 1} \right)m + 1 + w\;max\;(\delta ,\;0)} \hfill \\ \end{array}$$

Evidence about `momentum of approval'

#### Beliefs about groups

In the model beliefs about each group are represented in the form of a beta distribution. While this belief distribution changes with social approval or disapproval, initial beliefs for each of the groups are set via free parameters *n*^*(0)*^, α^*(0)*^_*min*_ and α^*(0)*^_*max*_.

13 $$\begin{array}{*{20}l} {\alpha^{\left( 0 \right)}_{M } = \alpha^{\left( 0 \right)}_{min } + \left( {M - 1} \right)\left( {\alpha^{\left( 0 \right)}_{max } - \alpha^{\left( 0 \right)}_{min } } \right)/\left( {n - 1} \right) } \hfill \\ {n^{\left( 0 \right)}_{M } = n^{\left( 0 \right)} } \hfill \\ {\beta^{\left( 0 \right)}_{M } = n^{\left( 0 \right)}_{M } - \alpha^{\left( 0 \right)}_{M } } \hfill \\ \end{array}$$

where α^(0)^_min_ is the α for the least accepting group, α^(0)^_max_ is the α for the most accepting group, n is the number of groups, and M = 1, 2, 3 and 4 for group 1, 2, 3 and 4.

On each trial, beliefs decay via the following equations,. The decay rate (l_acc_ ) represents the assumption that participants have limited working memory and thus older observations will be replaced by newer ones.

14 $$\begin{array}{*{20}l} {\alpha_{t }^{\prime } = \left( {1 - \lambda_{acc} } \right) \alpha_{t - 1 } + \lambda_{acc} } \hfill \\ {\beta_{t }^{\prime } = \left( {1 - \lambda_{acc} } \right) \beta_{t - 1 } + \lambda_{acc} } \hfill \\ {n_{t } = \alpha_{t }^{\prime } + \beta_{t }^{\prime } } \hfill \\ \end{array}$$

where l_acc_ is the decay coefficient for beliefs about groups.

During initial development on the discovery data only, it was found that while values of *n*^*(0)*^, α^*(0)*^_*min*_ and α^*(0)*^_*max*_ (13) were needed in order to set initial values, if all were set as free parameters, their values were underspecified or poorly recoverable. To improve parsimony and recoverability, *n*^*(0)*^ was turned from a free parameter into a value set by *l*_*acc*_:

15 $$n^{\left( 0 \right)} = 2 + 1/ \lambda_{acc}$$

This captures the intuition that the amount of (notional) data underpinning initial beliefs was comparable to the amount of (observed) data retained at any one time later.

After (14), i.e. decay of existing beliefs, occurs, beliefs are then updated, which will now be described in (16) below. Together, these two sets of equations form the full update policy: decay of existing beliefs (14) followed by updating of beliefs based on recent feedback, i.e. outcomes (16). Beliefs about a specific group got updated if feedback, i.e. approval or disapproval, from that group is encountered (*e*), and remained unchanged if feedback was not encountered (*ne*).

16 $$\begin{array}{*{20}l} {\alpha _{t}^{{\left( {ne} \right)}} = \alpha ^{{\left( {ne} \right)_{{t - 1}}^{\prime } }} } \hfill \\ {\beta _{t}^{{\left( {ne} \right)}} = \beta ^{{\left( {ne} \right)_{{t - 1}}^{\prime } }} } \hfill \\ {\alpha _{t}^{{\left( e \right)}} = \alpha ^{{\left( e \right)_{{t - 1}}^{\prime } }} + o_{t} } \hfill \\ {\beta _{t}^{{\left( e \right)}} = \beta ^{{\left( e \right)_{{t - 1}}^{\prime } }} + 1 - o_{t} } \hfill \\ \end{array}$$

where the outcome of a trial o_t_ = 1 on approval and o_t_ = 0 on disapproval.

#### Approval predictions

Once beliefs about groups were calculated, they were used to predict whether a certain group ‘approved’ or ‘disapproved’ of the participant (Fig. 1).

17 $$\pi_{L} = (1 + exp\left( { - \left( {G_{acc} + B} \right)/T} \right))^{ - 1}$$

where $\pi_{L}$ is the probability of the participant predicting approval. T is decision temperature, the magnitude of difference between approval probability G_acc_ = $\frac{\alpha }{n}$ and the indifference point (0.5) needed to increase the probability of predicting approval by a certain amount—an intuitive way of understanding this is that increasing decision temperature increases the randomness of one’s choices.

B is a free parameter which represents bias. This is a positivity bias (sometimes referred to as a self-serving bias) which captures the “extra credit” that people give themselves. Individuals with a higher positivity bias would thus be more likely to predict social approval, even in the absence of evidence that this is indeed likely.

The prediction the participant makes (approval prediction) is given by the binomial distribution *X* ~ *Bin*(1, p_L_).

### Beliefs about approval and generation of self-esteem ratings

We now turn to the generative model of momentary self-esteem. First, prediction errors for beliefs about groups are calculated upon encountering social approval or disapproval (Fig. 2b).

18 $$\begin{array}{*{20}l} {\delta = o_{t } - \alpha^{\left( e \right)^{\prime} } / n^{{\left( e \right)^{\prime } }} \Rightarrow } \hfill \\ {\delta^{ + } = \beta / n^{{\left( e \right)^{\prime } }} } \hfill \\ {\delta^{ - } = - \alpha / n^{{\left( e \right)^{\prime } }} } \hfill \\ \end{array}$$

where positive prediction errors d + occur on approval (o_t_ = 1), negative prediction errors d- occur on disapproval (o_t_ = 0), and the group-specific expectations are taken from Eq. (16).

Similar to beliefs about groups, beliefs about approval (*S*) are represented in a beta distribution with parameters a and b. The prediction errors above in (18) are then incorporated into the ‘beta-belief’ about the self via (12):

12 $$\begin{aligned} \alpha_{t}^{{S}} & = (\alpha_{{t-1}}^{{S}}-1)\varsigma + 1 + w\, max\, (\delta,0) \\ \beta_{t}^{{S}} & = (\beta_{{t-1}}^{{S}}-1)\varsigma + 1 + w\, max\, (\delta,0) \\ \end{aligned}$$

(repeated from Box 4 for clarity).where *w* weighs the prediction error and $\varsigma$ is a trial by trial belief decay term.

Self-esteem ratings are then generated as follows. First, a value is drawn randomly from the beta distribution generated from the parameters in Eq. (12) above. Then, a sigmoidal response function translates this value into self-esteem ratings:

19 $$SE = \frac{1}{{1 + \left[ {\left( {1 - P_{acc} } \right)/\left( {P_{acc} B} \right)} \right]^{m} }}$$

where P_acc_ is the randomly-drawn value from Eq. (12) and m represents the sensitivity of self-esteem changes. The higher the sensitivity (m), the more one’s momentary self-esteem fluctuates in response to one’s beliefs about approval. B represents the participant’s shift or bias. It captures the participant’s baseline self-esteem, i.e. the level of self-esteem which momentary fluctuations are built on. The higher the bias, the higher their baseline self-esteem.

Initial self-esteem levels are set by $P_{acc} = \frac{{\alpha_{1} + \alpha_{2} + \alpha_{3} + \alpha_{4} }}{{ n_{1} + n_{2} + n_{3} + n_{4} }}$ and applying Eq. (19).

An overview of the free parameters of the belief model is given in the Supplement (S4).

### Model fitting and comparison

An overview of the model space used for comparisons is given in Table 5.

**Table 5:** An overview of the model space being compared.

Associative model	Self-esteem depends on the history of social prediction errors (when outcomes violate expectations). Expectations are updated by a Rescorla–Wagner rule^6,46^
Associative model (dual learning rates)	Similar to the associative model, but with different learning rates for social approval and disapproval
Associative model (dual learning rates adnd separate term for expectations)	Similar to above, but with a separate term for summed expectations about approval in addition to summed social prediction errors
Associative model (dual learning rates and fixed positive bias)	Similar to the associative model (dual learning rates), but with a fixed bias towards predicting approval despite contrary expectations
Associative model (with competence)	Similar to the associative model, but with the addition of a term which captures competence at predicting social approval or disapproval, irrespective of feedback valence
Associative (sigmoid) model	Similar to the associative model but with a sigmoid response function instead of a Gaussian term
Belief model	Beliefs, formalised as beta distributions, are used to calculate social prediction errors to update self-esteem levels

Maximum Likelihood Estimation^48^ (MLE) was used to determine which parameters of the model provide the best fit to a certain participant’s data, and the Bayesian Information Criterion (BIC), which accounts for both likelihood and model complexity to prevent overfitting, was used for model comparison. The BIC for each participant was calculated by the following equation, where *n* is the number of data points (in this case number of self-esteem and prediction ratings), *k* is the number of free parameters used to fit, and L is the maximum likelihood (or maximum likelihood density, for continuous measures),

20 $$BIC = ln\left( n \right)k - 2ln\left( L \right)$$

In addition to BIC, mean squared error over self-esteem ratings were used to examine how well models described the data. Models were written in the programming language R^49^.

Models were checked for appropriate parameterization and behaviour, synthetic data with similar features to participant data was generated and re-fitted, and parameter recovery checks were conducted. Then, parameters were fit to participant data. Fitting parameters to data was then done with the non-linear minimisation (*nlm*) function from R. To avoid local optima, each participant was fitted using initial conditions from a grid of 129 sets of parameters. For each of the 129 fitting attempts per participant, the *nlm* function was applied to find the maximum-likelihood.

## Supplementary Information

Supplementary Information.

## Data Availability

The data analysed in this study are available from the first authors. The data from the ‘confirmation’ sample, which was collected under the Neuroscience in Psychiatry Network Project, is also available from https://openNSPN@medschl.cam.ac.uk.

## Code availability

Analysis code for all results obtained here is available from https://github.com/alexisaylow/selfeval and further support on its usage is available from the first authors.
