# Supplementary material for: Self-esteem depends on beliefs about the rate of change of social approval
Source: Sci Rep. 2022 Apr 22;12:6643. doi: 10.1038/s41598-022-10260-6 (PMC9033861; doi:10.1038/s41598-022-10260-6)
Supplement: Supplementary file 1 — Supplementary Information. [file 41598_2022_10260_MOESM1_ESM.pdf]

Supplementary information for

Self-Esteem depends on beliefs about the rate of change of social approval

Alexis An Yee Low[1*#], William John Telesfor Hopper[2#], Ilinca Angelescu[3], Liam Mason[3,4], Geert-Jan Will[5] and Michael Moutoussis[1,3]

[1] Wellcome Centre for Human Neuroimaging

[2] Paris Brain Institute, Paris, France

[3] Max Planck UCL Centre for Computational Psychiatry and Ageing Research, University College London, London UK

[4] Research Department of Clinical, Educational and Health Psychology, University College London, London, United Kingdom

[5] Department of Clinical Psychology, Utrecht University, Utrecht, the Netherlands

* Corresponding Author: an.low.16@ucl.ac.uk

# Joint first authors

Supplementary Note

Why the Sum-of-prediction-errors is an estimate of momentum

First, let us clarify the rich meaning of the concept of momentum. When a quantity has ‘momentum’, we mean two things. First, that momentum is proportional to the rate of change in time of another quantity, which in turn can be understood as ‘where something is’, a literal or metaphorical ‘position’. This rate of change includes information of the direction in which the ‘position’ changes, and is hence a velocity rather than a mere speed: momentum $\boldsymbol{p} \sim \mathrm{d}\boldsymbol{x}/\mathrm{dt} = \boldsymbol{v}$. In our case, the ‘position’ is the social position of an individual. We expressed this social position as the level of approval in belief models, or as an affective value of the social group in associative models. However, momentum formally has a second, so-called inertial aspect. It measures how difficult it is to change this ‘velocity’. Greater force is required to change the velocity by a given amount if the momentum is greater. In many contexts, this inertial aspect can be ignored as a fixed proportionality constant, but in our case, it has a most interesting interpretation. If ‘force’, in our case, is the evidence that a person encounters, then the ‘inertia’ naturally becomes the inverse of the learning rate (associative models) or simply the precision or confidence of beliefs (inferential models). Here, the more precise or confident the beliefs the less they will shift given a certain amount of evidence. Similarly in an associative context, the greater the inverse of the learning rate, the lesser the impact of a given prediction error.

We now turn to the weighted sum of prediction errors as a measure of momentum, namely, as proportional to the rate of change of estimated social position. First, we note that if approval were not probabilistic and a single approval even accurately represented the underlying approval rate, then a single prediction error would simply measure how much value or belief has changed since the last observation, and would thus be proportional to this rate of change. In the case of noisy feedback, to estimate the true rate of change we average over several events, but we recency-weigh them as this rate of change will itself typically change with time, so prediction errors will lose their representativeness the further they are in the past. Finally, we demonstrate this process in the following figure. We can see that mean Self-evaluation (red) closely tracks a smoothed estimate of the gradient of value (yellow).

Supplementary Figure S1.

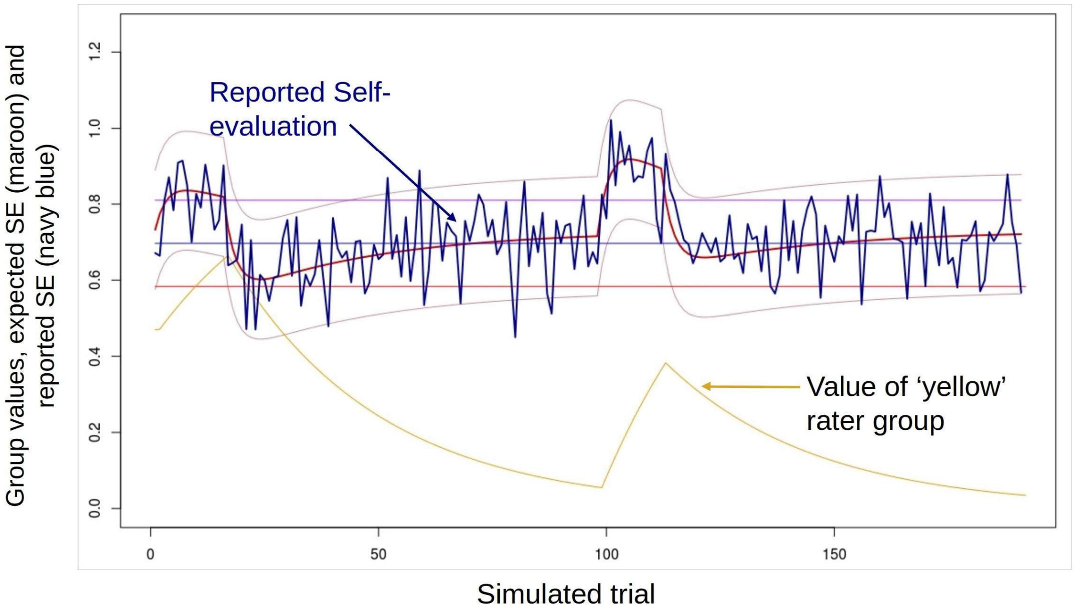

Demonstration of how state-Self-esteem resembles the temporal gradient, or rate of change, of the value associated with acceptance in the exponential-kernel model. For the purposes of demonstration, the agent, who has parameters characteristic of real participants in the discovery group, only encounters raters with 15% approval rate, with trials grouped as all positive (trials 1-15), all negative (16-100) etc. It can be seen that the simulated mean SE (maroon) and reported SE (blue), track the radent of the yellow line, showing step-like increases and reductions when the yellow curve switches from increase to decrease, etc.

Supplementary Table S2. Model comparison of baseline vs. two-learning-rate model for the test (subclinical) sample.

Model	Sum BIC	Mean BIC	Median BIC
Original	**-1378**	**-22.6**	**-23**
2LR	-794	-13.0	-27
2LR + separate term for expectations	-813	-13.3	-10
2LR + fixed positivity bias	268	4.4	15
Competence-Acceptance (best, including separate expectation term)	-1280	-20.6	-20

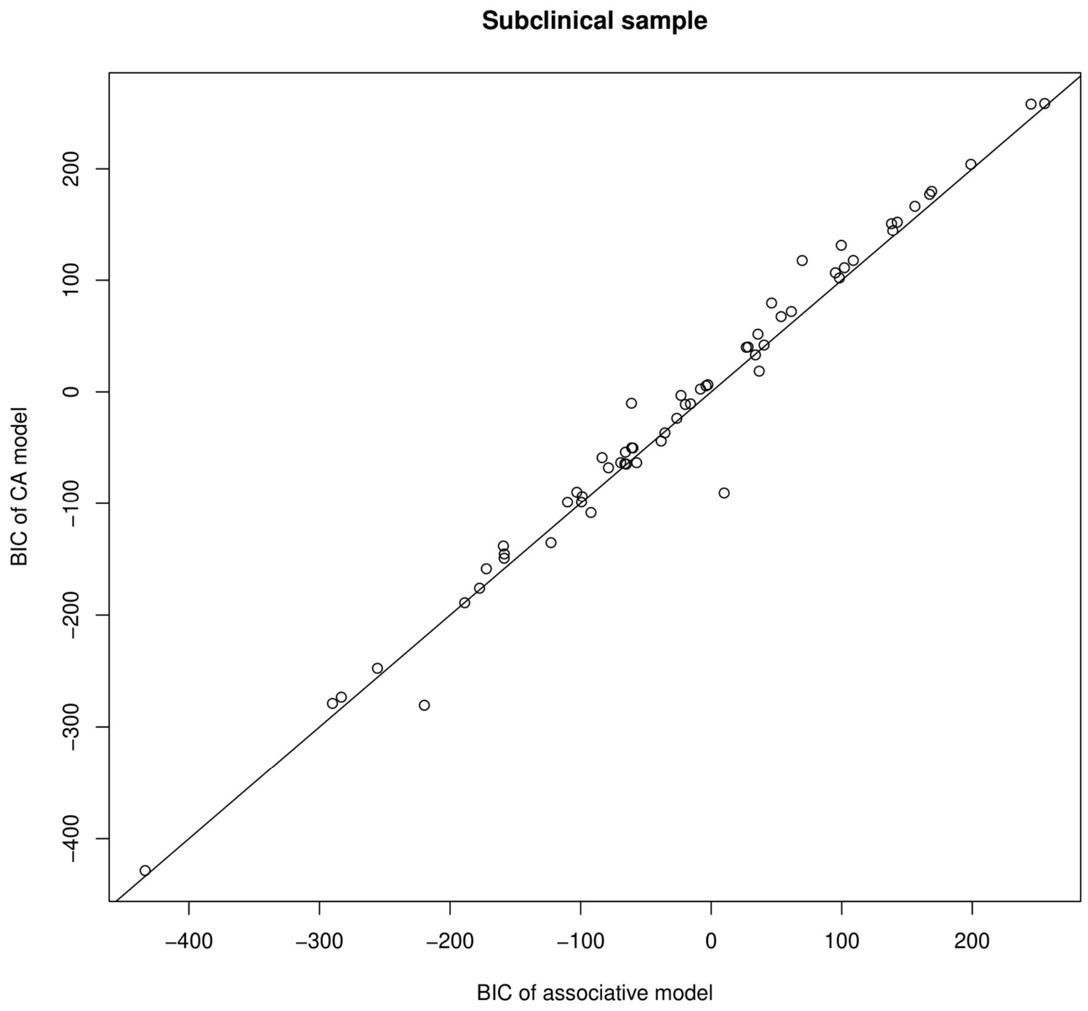

Supplementary Figure S3. The BIC per participant for the baseline model was worse for the Competence-Acceptance model for most participants, due to its higher complexity. See Table 1 for summary statistics. Just two participants appear to be much better fit by the Competence-Acceptance model.

Supplementary Methods: Modelling

Supplementary Table S4. Symbols, definitions and ranges for the free parameters for all models.

Symbol	Range	Definition
**Associative model - Original**		
w_0	**0-1**	Baseline self-esteem of participant
**ESV_1**	**0-1**	Initial Maximum Expectation (i.e. of rater group 1, i.e., the rater group highest in approval rate)
**ESV_4**	**0-1**	Initial Minimum Expectation (i.e. of rater group 4, i.e., the rater group lowest in approval rate)
η	**0-1**	Learning rate for updating expectations. The higher the learning rate, the more weight an agent places on the prediction errors they receive. This means that prediction errors have a greater impact on learning.
γ	**>0**	Forgetting factor - quantifies decay of effect of history of social feedback on self-esteem to model that recent events have a greater impact than earlier events.
w_1	**>0**	Weight of prediction errors on momentary self-esteem
τ	**>0**	Decision temperature - controls how large a difference in a decision-variable is needed to motivate behaviour change. Technically, the difference between expectation and the indifference point for a specific change of the probability of predicting approval. An intuitive way to understand it is that the higher the temperature, the more difficult to shift choice away from indifference, so the more random the choice.
**B**	**0-1**	Positivity bias that captures a tendency to persist in predicting approval despite low expectations

ϵ	>0	Noise term for capturing the assumption that participant's reported self-esteem varied around their underlying self-esteem according to a gaussian distribution
Associative model - Separate term for expectations		
w_{EV}	>0	Weighting factor for separate expectations term
Associative model - Valenced Learning Rates		
η_{pos}/η_{neg}	0-1	Learning rate when participant's prediction error was positive (resp. negative)
Associative model - Competence-Approval		
w_3	0-1	Weighting factor for capturing how much a participant's self-esteem is affected by their competence versus their approval by raters, with a value of 1 (resp. 0) meaning solely dependent on approval (resp. competence).
Belief-based model		
$n^{(0)}$	>0	Number of observations one's beliefs about groups are based on
$a^{(0)}_{min}$	>0	α for least accepting group
$a^{(0)}_{max}$	>0	α for most accepting group
λ_{acc}	0-1	Decay rate for beliefs about groups
T_{pred}	>0	Decision temperature for approval predictions (similar to above)
B_{pred}	$\mathbb{R}$	Bias term for approval predictions. Similar to the positivity bias above, it captures the "extra credit" that people give themselves. Individuals with a higher positivity bias would thus be more likely to predict social approval, even in the absence of evidence that this is indeed likely.

$\boldsymbol{w}$	**>0**	Weight placed on updates to beliefs about approval
$\boldsymbol{\zeta}$	**0-1**	Retention rate of beliefs about approval i.e., 1 - decay rate
$\boldsymbol{m}$	**>0**	Sensitivity (slope) of sigmoid response function. The higher the sensitivity, the more one's self-esteem changes in response to changes in beliefs about approval.
$\boldsymbol{B}$	**>0**	Shift (bias) of sigmoid response function. This captures the participant's baseline self-esteem. For example two participants may both expect 50% approval, on average, but report SE of 0.1 vs. 0.9. The latter would be equipped with a much higher *B*.
